# Medication Handling and Storage among Pilgrims during the Hajj Mass Gathering

**DOI:** 10.3390/healthcare9060626

**Published:** 2021-05-24

**Authors:** Saber Yezli, Yara Yassin, Abdulaziz Mushi, Bander Balkhi, Andy Stergachis, Anas Khan

**Affiliations:** 1 The Global Centre for Mass Gatherings Medicine, Ministry of Health, Riyadh 12341, Saudi Arabia; yara@yassin.com (Y.Y.); abhmushi@moh.gov.sa (A.M.); anaskhan@ksu.edu.sa (A.K.); 2 Department of Clinical Pharmacy, College of Pharmacy, King Saud University, Riyadh 11451, Saudi Arabia; bbalkhi@KSU.EDU.SA; 3 School of Pharmacy, University of Washington, Seattle, WA 98195, USA; stergach@uw.edu; 4 School of Public Health, University of Washington, Seattle, WA 98195, USA; 5 Department of Emergency Medicine, College of Medicine, King Saud University, Riyadh 11451, Saudi Arabia

**Keywords:** Saudi Arabia, medication, storage, drug stability, knowledge, health education

## Abstract

We aimed to investigate the knowledge and practices of Hajj pilgrims regarding medication storage and handling during the Hajj mass gathering. In this cross-sectional study, adult pilgrims from 30 countries were interviewed using a structured questionnaire during the 2019 Hajj. The study enrolled 1221 participants with a mean age of 50.8 years (SD = 12.5, range = 18–98) and male:female ratio of 1.7:1. Most pilgrims were literate, 50.4% had a university or higher education, and 38% reported at least one underlying health condition. Most pilgrims reported receiving education regarding the proper way to store their medication during Hajj, mainly from physicians (73.7%) and pharmacists (39.4%). Although 68.2% of pilgrims had good knowledge regarding medication storage and the potential effect of inappropriate storage conditions on medications and health, inadequate knowledge and poor practice were identified among some. Level of education, having an underlying health condition and receiving health education on mediation storage were independently associated with good knowledge. Most pilgrims took their medications with them during Hajj, although storage and handling of their medication also varied depending on the stages of their Hajj pilgrimage journey. Improving Hajj pilgrims’ awareness and knowledge about appropriate storage and handling of their medications are beneficial in reducing the risk of associated adverse health outcomes, both during Hajj and beyond the mass gathering.

## 1. Introduction

Medicinal products require appropriate storage conditions to ensure their quality, safety and efficacy. These conditions include the characteristics of the storage area (e.g., adequate size, clean, safe, and dry), as well as environmental factors, such as exposure to humidity, light and temperature. Extremes in these environmental factors may cause chemical, physical, and microbiological changes, especially if medicinal products are stored outside of their original packaging or stored near food and/or chemicals [1,2]. Temperature, in particular, is a key variable in drug stability [2,3]. Humidity and light are additional environmental factors that can affect medications. If medications are significantly degraded, this may affect their clinical effectiveness, which may result in changes in dosage, storage conditions, or how often they need to be replaced [3,4]. For most drugs, manufacturers assure a potency of 90–110% for a specified period, but only if stored as recommended [2]. The storage requirements for medicine can be found on the medication label itself and/or on the product package insert. Patients should comply with these storage instructions, to avoid potential adverse consequences, such as poisoning, exacerbation of underlying health conditions and death. Medicines that lose their effectiveness can also lead to waste and financial loss.

The Hajj religious mass gathering is attended annually by over 2 million Muslim pilgrims from around the world. Many Hajj pilgrims are elderly and have underlying health conditions that are managed by medications [5]. It is expected that these pilgrims will bring such medications with them on the pilgrimage [6]. In addition to drugs for chronic conditions, pilgrims also bring other types of medications with them for the pilgrimage in case they are needed, including analgesics, antipyretics and antibiotics [6,7]. Within the Kingdom of Saudi Arabia (KSA), pilgrims also access medications through physicians and pharmacists and from other pilgrims. They can also acquire medication via their Hajj medical missions. The latter are deployed by many countries to Saudi Arabia during the Hajj season to provide basic curative medical services and other related health services to pilgrims from their own countries. Many factors make the appropriate storage and handling of medications challenging during Hajj, particularly for pilgrims with chronic conditions who need to take their medication on a regular basis or for those using temperature-sensitive medications that need to be kept at cool/cold temperatures. The pilgrimage takes place in Makkah, Saudi Arabia, characterized by a hot desert climate where daily temperatures exceed 25 °C most of the year and can reach up to 50 °C, particularly on summer days [8]. The Hajj journey involves travel to and within KSA by planes, cars, buses and trains, and may include extended hours of flight and long transit and waiting times. The Hajj rituals involve numerous outdoor physical activities, sometimes in very hot weather conditions, and the movement, often on foot, between Makkah and the holy sites of Mina and Arafat [9]. Many pilgrims also travel to the city of Madinah as part of their Hajj journey. 

Studies report that a large proportion of the public does not have adequate information and appropriate practice regarding medication use, handling, storage and disposal [10,11,12,13]. Similar observations were previously reported among Hajj pilgrims [7,14]. The inadequate handling, storage and disposal of medication pose a serious health and safety risk. This can be, for the patients and their immediate contacts, through the use of less potent, compromised or toxic drugs or via accidental poisoning, as well as for the community at large through environmental contamination by active pharmaceutical ingredients [12,15,16]. Given the diversity among Hajj pilgrims in terms of language, culture and beliefs, health literacy and educational and health system backgrounds, there is a need to evaluate knowledge and practice regarding medication storage and handling among pilgrims from various nationalities. Findings from such an investigation would help develop the appropriate policies and health education and awareness campaigns to improve pilgrims’ knowledge and practice and reduce the risk of adverse health outcomes. Here, we evaluate Hajj pilgrims’ knowledge and document their practice in relation to medication storage and handling. 

## 2. Materials and Methods

### 2.1. Study Setting, Design and Population

A cross-sectional descriptive study, using a convenience sampling technique, was conducted among pilgrims in Makkah, Saudi Arabia, during the 2019 Hajj season (from 14–19 August 2019). The minimum sample size estimated for the study was 1062, based on a 3% margin of error, a confidence interval (CI) of 95%, an approximate pilgrims’ population of 2 million, as well as an expected 50% response rate to most of the core questions. After adjusting for a projected 10% attrition, the estimated final sample size for the study was at least 1200 participants. 

### 2.2. Data Collection Tool and Scoring System

Data was collected via face-to-face interviews using an anonymous questionnaire developed based on available literature and tailored for the Hajj context. The questionnaire was initially reviewed by two experts for any suggestions or amendments, then piloted among 20 pilgrims and finalized. The questionnaire collected basic demographic and health data, as well as information regarding pilgrims’ knowledge and practice relating to medication storage and handling. The questionnaire was divided into three sections, with questions regarding: (1) demographics (including age, gender, nationality, literacy, education level, and underlying health conditions), (2) general medication storage and handling (3) medication storage and handling during the Hajj pilgrimage. A previously described scoring system [17] was used to score the knowledge responses. Overall mean knowledge scores, ranging from 0 to 1, were calculated, and pilgrims with scores > 0.75 were considered to have a good level of knowledge. 

### 2.3. Statistical Analysis

Descriptive statistics (e.g., mean and standard deviation (SD)) were computed for quantitative variables, while frequencies and percentages were calculated for categorical variables. The association between categorical variables was evaluated by Chi square test. Odds ratios with 95% CIs were calculated to assess the presence and degree of association between the dependent versus independent variables. Variables with *p*-values < 0.05 at the bi-variable analysis were taken for multivariate analysis. SPSS 22.0 (SPSS Inc., Chicago, IL, USA) software was used for all analyses.

### 2.4. Ethics and Confidentiality

The study was approved by the King Fahad Medical City Ethics Committee and the Institutional Review Board (IRB Log: 19-407E). All participants were informed about the study by trained researchers and gave verbal consent. The study questionnaire was anonymous and did not include any identifiers or personal information of the participants. 

## 3. Results

### 3.1. Characteristics of the Study Population 

The study enrolled 1221 participants from 30 countries, mainly from the Middle East and North Africa and South Asia (Table 1). The countries most represented were Nigeria (15.4%), India (12.8%), Indonesia (10.5%) and Pakistan (9.1%). Pilgrims had a mean age of 50.8 years (SD = 12.5, range: 18–98) and 63.3% were male. Most pilgrims (90.2%) were literate, and half had a university or higher education. Nearly 38% of participants reported at least one underlying health condition, most commonly diabetes (24.2%) and hypertension (20.9%) (Table 1). 

### 3.2. Medication Storage Knowledge and Practice among Hajj Pilgrims

**Figure 1:**
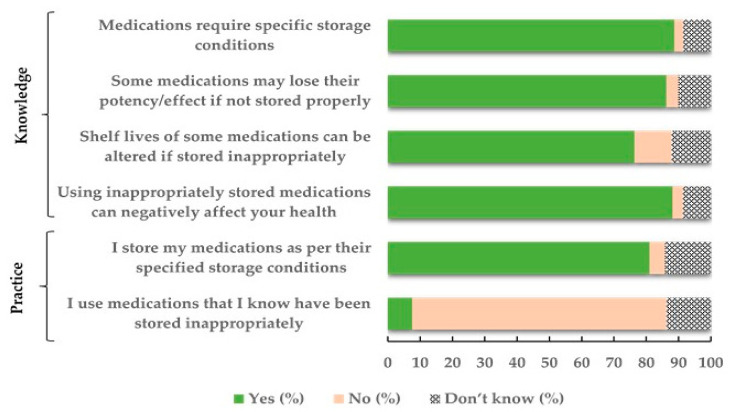
Hajj pilgrims’ knowledge and practice regarding medication storage.

Nearly 78% of pilgrims reported receiving education regarding the proper way to store their medication during Hajj. Most of this education was in their country of origin (95.5%), while only 8.1% received such information in KSA. The most common sources of knowledge regarding appropriate medication storage among pilgrims were physicians (73.7%), followed by pharmacists (39.4%), the medication label itself (28.2%), and other sources (6.6%), such as the internet and family members. Most pilgrims had basic knowledge regarding medication storage and the potential effect of inappropriate storage conditions on medications and on health (Figure 1). Still, 4.7% reported not storing medications properly and 7.6% would use medications that they know were stored inappropriately. 

The overall mean medication storage knowledge score among pilgrims was 0.84 (SD = 0.29), with 68.2% of participants considered to have good knowledge according to our criteria (mean score > 0.75). Level of education, having an underlying health condition and receiving health education on medication storage were independently associated with good knowledge (Table 2). Pilgrims with an underlying health condition or those having received health education on medication storage were more than twice as likely to have good knowledge; OR = 2.7 (95% CI = 1.4–5.0, *p* = 0.001) and OR= 2.3 (95% CI = 1.3–4.0, *p* = 0.002), respectively. Compared with pilgrims with no formal education, pilgrims with a university/higher education were more than 24 times more likely to have good knowledge of medication storage (OR = 24.4, 95% CI = 10.1–58.9, *p* < 0.0001).

### 3.3. Medication Sources and Handling during the Hajj

Of all participants, 534 (44.4%) reported using medication during Hajj, including 96% of those with underlying health conditions. These pilgrims were questioned further regarding the sources, handling and storage of their medication during the pilgrimage (Table 3 and Table 4). Most pilgrims (91.6%) reported using 1–4 medications, while the rest reported using 5 or more medications at the same time (polypharmacy) (Table 3). Nearly 87% of pilgrims reported acquiring their medication from outside KSA and 60.2% obtained medications from within KSA. Among pilgrims with underlying health conditions, 93.6% brought medication with them. In KSA, most pilgrims (78.8%) acquired their medication through a physician, 36.6% via a pharmacist and 2.5% from family and friends. During Hajj, most pilgrims reported the self-handling of their medication (81.2%) and carrying medication with them during the Hajj rituals (71.5%). The medical mission was the second most common body that handles pilgrims’ medications during Hajj (Table 3). In relation to medication remaining post-Hajj, most pilgrims (83.9%) reported that they would take it back home with them, 15.3% would discard it, and a minority (1.1%) would donate it or leave it with their medical mission.

### 3.4. Medication Storage during Hajj

During their Hajj journey, most pilgrims (>80%) stored their medications with them (Table 4). The majority of the rest stored medications with their Hajj medical missions, while a minority (<2%) used shared storage with other pilgrims or used other means (mainly storage with family members). The proportion of pilgrims who stored their medications with medical missions was significantly higher in the holy sites of Mina and Arafat compared to when pilgrims were staying in Makkah or Madinah (26.4% vs. 17.9%; *p* = 0.001).

## 4. Discussion

The appropriate storage of medications, within recommended temperature ranges, is vital to maintaining their integrity, as well as contributing to patients’ safety. This is particularly relevant during travel and in hot climate settings; such is the case during Hajj, where recorded temperatures during the event ranged between 37 °C and 45 °C [18]. In such settings, the appropriate storage of medication can be doubly challenging. Certain medications (e.g., insulin, glucagon, some antibiotics, certain reconstitute drugs and some eye drops) are required to be stored in refrigerators as per manufacturers’ instructions [19]. Hence, pilgrims need to ensure the availability of a refrigerator or an alternative cold storage facility during Hajj. In addition, medication required to be stored at “room temperature” may still be affected given the high environmental temperatures during the pilgrimage. Extreme temperatures cause the degradation of medications, which may change their pharmacological characteristics, reduce their potency and efficacy or result in compromised or toxic products, leading to adverse health outcomes [1,2,3,4,20]. Consequently, the appropriate handling and storage of medication among pilgrims during Hajj are of public health importance.

In the current study, underlying health conditions were common among pilgrims, particularly diabetes and hypertension, which is in line with other reports from Hajj [5]. Pilgrims with such conditions often take medication to manage their illnesses which they bring with them from outside KSA; although not always in sufficient quantities [6,21]. In a 2017 study, over half of pilgrims with chronic conditions brought their medications on Hajj, but only 12% reported having enough quantities covering their stay at the event [6]. Pilgrims should be encouraged to bring their chronic illnesses medications with them in sufficient amounts to ensure that they do not run out and to avoid deterioration of their underlying health conditions [21]. On the other hand, bringing unnecessary medication in excessive amounts can promote misuse and self-medication, as well as creating issues regarding the handling, storage and disposal of such drugs. This is evident in the case of antibiotics, where self-medication and misuse can impact health outcomes and increase the risk of antibiotic resistance, while inappropriate storage can render these medications toxic. Studies among Hajj pilgrims found that up to 60% of Hajj pilgrims questioned brought antibiotics from their home country to KSA, and these drugs were used inappropriately for prophylaxis and the treatment of non-bacterial infections [7,22].

Studies report that a large proportion of pilgrims are unaware of the appropriate use, handling, storage and disposal of medicines [7,14]. In one study, only 42% of pilgrims questioned understood the proper way to use their medication in terms of frequency, dose, administration method, side effect monitoring and interaction [14]. In the same study, only 38.6% of respondents reported knowing how to correctly store their medication and merely 12% knew that all medicines have to be kept at specific temperature ranges. In the current study, pilgrims had good basic knowledge regarding medication storage and the impact of inappropriate storage on medication efficacy, shelf life and on health. The high proportion of pilgrims with a university or higher education among the study population may have contributed to this observation. Additionally, most respondents claimed to store their medications according to the recommended storage conditions and not to use medications stored inappropriately. While these results are encouraging, interventions are needed to address lack of knowledge and poor practices among the remainder of the pilgrims questioned.

Level of education, having an underlying health condition, and receiving health education on mediation storage were independently associated with good knowledge. Level of education is associated with better knowledge of medications, their handling and storage [23,24,25,26]. Being educated enables patients to understand and practice the written and oral guidelines and instructions of medication use, storage and handling better than those who are illiterate. Similarly, educational interventions have been shown to improve patients’ knowledge of their medication, including appropriate storage [27,28]. Likewise, pilgrims with underlying health conditions are more likely to be taking medications regularly and for longer periods of time compared to others. As such, it is expected that these pilgrims have better knowledge regarding medication handling and storage than those without underlying health conditions, who may only periodically take medications.

Physicians and pharmacists were the main sources of information regarding medication storage among pilgrims. Similar observations were reported among Hajj pilgrims and in general populations [10,14]. This is not surprising, given that these healthcare providers are the primary sources of medication prescribing and dispensing. However, a small proportion of pilgrims reported obtaining such information from other sources, such as the internet and family members [14]. One study found that 52–67% of Hajj pilgrims questioned obtained information regarding medication from non-medical sources (e.g., the internet, magazines, TV, traditional healers and friends and family). These are not always reliable sources of medical information, and pilgrims should be aware of the risk of following directions on how to use, store and dispose of medication obtained exclusively from such sources. Physicians, pharmacists and other healthcare providers have a captive audience at the point of prescribing or dispensing, hence the opportunity to effectively educate patients regarding the use, storage and eventual disposal of any leftover medications. Healthcare providers should also verify the validity of patients’ health information and to counsel patients and provide them with the needed information regarding their medications. As seen in the current study and others [6,7], many pilgrims bring medication from their own countries, which may have different brand names and various dosage forms and strengths [29] Therefore, healthcare providers have the added duty to ensure that pilgrims are aware of these factors, especially if pilgrims are prescribed similar medications during Hajj.

In addition to healthcare providers, the medication label and product insert are also important sources of information on medication use and storage. Only 28.2% of pilgrims accessed information on medication storage from the medication label in the current study. Studies have shown that not all medication users consult the medication label or the package insert for information [13,14,15,16,17,18,19,20,21,22,23,24,25,26,27,28,29,30]. In one study among Hajj pilgrims, only 54.2% reported that they do read the information on medications’ labels before using them. Moreover, a significant proportion also reported having troubles reading labels for medications supplied for them during Hajj, which was linked to pilgrims’ level of education. This is supported by the fact that lower literacy is associated with misunderstanding the instructions on prescription medication labels [31,32]. As such, policymakers should consider evidence-based adjustments to medication labels for optimum format and content for readability and understanding by patients during Hajj and to ease communication with and comprehension by the end user [33].

While most pilgrims stored their medications with them during Hajj, storage practices varied depending on the stages of the pilgrimage journey. The proportion of pilgrims who stored their medication with their medical missions was significantly higher in the holy sites of Mina and Arafat compared to when pilgrims were staying in Makkah or Madinah. This observation may be linked to the availability of storage facilities, including cold storage (e.g., refrigerators), at accommodations in both locations. During their stay in Makkah and Madinah, pilgrims reside mainly in standard hotels, with potentially more storage space and access to refrigerators. On the other hand, in the holy sites of Mina and Arafat, pilgrims reside in large purposefully built tents, with limited storage and access to cold storage facilities. Therefore, in these locations, more pilgrims stored their medications with their medical missions, which may have access to better storage facilities/conditions. We also note that only a small minority of pilgrims chose to store their medication in shared storage facilities during Hajj. This is reassuring, as such practice could lead to the mixing of medication and medication error.

In our study, 15.3% of pilgrims declared that they would discard medication remaining post-Hajj. Pilgrims should be aware of the appropriate way to dispose of their unwanted or expired medication [34]. Studies report that a large proportion of patients do not receive information about proper medication disposal [12]. Often, unwanted or expired medications are disposed of via the sink, thrown in the garbage, or flushed down the toilet [12,35], thus, posing risks to the community through environmental contamination by active pharmaceutical ingredients [15]. In addition, some medications are injected using syringes or needles, which are a biomedical hazard. If not disposed of correctly, these could lead to needle-stick injuries and contracting blood-borne diseases [36]. Thus, these sharp medical supplies must be disposed of in a designated sharps container and not placed directly into the trash [36,37,38].

Education is an important factor in improving patients’ knowledge of their medications, including storage conditions [27,28,39]. Educating pilgrims regarding appropriate handling and storage of their medications, including during the Hajj journey, is crucial to improve their knowledge and practice, both within and outside the Hajj context. Health education ought to start at the country of origin and continue during pilgrims’ stay in KSA and should be led by physicians and pharmacists, as these are the main sources of medication among pilgrims. Moreover, literate pilgrims should also be encouraged to consult the medication label and product insert for further information regarding their medication. Given the diversity in pilgrims’ level of education and health literacy, it may also be beneficial to identify pilgrims with limited health literacy and offer them tailored medication counseling that fits their needs [40]. Medical missions have a significant role to play in providing education on medication and in ensuring good practice among pilgrims, especially at Hajj holy sites where storage facilities are limited. This is because pilgrims find it easier to obtain and understand information about medications from healthcare providers from their own medical missions, due to ease of communication [14].

Our study has some limitations. Although we recruited a large number of pilgrims from different countries, the sampling methodology and the potential for volunteer bias may limit the generalizability of the findings. Additionally, data were collected using a questionnaire; therefore, responses obtained were prone to information bias. In addition, practice was self-reported and not observed. Finally, the study did not capture the exact number and type of medications used by pilgrims. As such, beyond polypharmacy, it was not possible to differentiate between pilgrims who were taking one medication or multiple medications. Additionally, the study did not report on the proportion, as well as knowledge and practice, of participants who were taking medication with specific storage requirements such as insulin. Future studies should investigate the knowledge, beliefs and behaviors of this subpopulation regarding medication storage and handling in Hajj.

In conclusion, this is the first large scale study among Hajj pilgrims to investigate their knowledge and practices regarding handling and storing their medications during the event. While most pilgrims had good basic knowledge, knowledge gaps and inappropriate practices were reported by some. Interventions to improve pilgrims’ knowledge and practice regarding their medication should ensure that: pilgrims do not bring unnecessary medications to Hajj; bring their essential medications in adequate quantity to cover their stay in KSA; and that they are aware of the storage conditions for their medications and the appropriate way to dispose of them. Awareness of weather conditions in Makkah and facilities available for pilgrims in Hajj, including access to healthcare, is important for pilgrims to prepare for the Hajj journey and to ensure that their medication will be stored and handled appropriately.

## Figures and Tables

**Table 1:** Characteristics of the study population.

Variable	*N*	%
**Total participants**	1221	
**Gender**		
Male	768	63.3
Female	445	36.7
**Age (Years)**		
Mean, SD (range)	50.8, 12.5 (18–98)	
<40	221	8.3
40–59	666	55.2
≥60	320	26.5
**Nationality**		
Middle East and North Africa *	431	35.8
South Asia	316	26.2
Sub-Saharan Africa	230	19.2
Southeast Asia	211	17.5
Other **	16	1.3
**Literacy**		
Yes	987	90.2
No	107	9.8
**Educational level**		
Universe/higher education	538	50.4
Secondary education	330	28.5
Primary education	138	11.9
No formal education	105	9.1
**Underlying Health conditions**		
No	715	62.4
Yes	430	37.6
Diabetes mellitus	277	24.2
Hypertension	239	20.9
Cardiovascular disease	34	3.0
Chronic kidney disease	5	0.4
Chronic lung disease	14	1.2
Chronic liver disease	4	0.3
Immunosuppressive illness	4	0.3
Cancer	5	0.4

* Including Turkey; ** USA, Canada, Australia and New Zealand, SD; standard deviation.

**Table 2:** Association between variables and good knowledge of medication storage among Hajj pilgrims.

Variable	*N*	OR	95% CI	*p*-Value	aOR	95% CI	*p*-Value
**Gender**							
Female	445	1					
Male	768	1.00	0.77–1.28	0.99			
**Age (years)**							
<40	221	1			1		
40–59	666	0.99	0.70–1.39	0.96	1.22	0.50–2.95	0.65
≥60	320	0.54	0.37–0.78	**0.001**	0.78	0.30–2.02	0.61
**Nationality**							
Sub-Saharan Africa	230	1					
Middle East and North Africa *	431	1.05	0.74–1.51	0.73			
Other **	16	1.15	0.36–3.71	0.80			
South Asia	316	0.47	0.33–1.03	0.08			
Southeast Asia	211	1.01	0.67–1.54	0.93			
**Literacy**							
No	107	1					
Yes	987	0.98	0.64–1.51	0.96			
**Educational level**							
No formal education	105	1			1		
Primary education	138	6.13	3.45–10.97	**<0.0001**	7.65	3.17–18.47	**<0.0001**
Secondary education	330	6.14	3.69–10.21	**<0.0001**	5.11	2.25–11.59	**<0.0001**
Universe/higher education	583	14.03	8.51–23.13	**<0.0001**	24.43	10.12–58.98	**<0.0001**
**Underlying health conditions**							
No	715	1			1		
Yes	430	0.75	0.58–0.97	**0.03**	2.72	1.47–5.03	**0.001**
**Education on medication storage**							
No	112	1			1		
Yes	383	1.94	1.26–2.98	**0.002**	2.37	1.38–4.05	**0.002**

* Including Turkey; ** USA, Canada, Australia and New Zealand; OR; odds ratio, aOR; adjusted odds ratio.

**Table 3:** Medication sources and handling during the Hajj pilgrimage.

Variable	Category	*N*	%
Pilgrims using medication during Hajj		534	44.4
Where did you acquire your medication?	Outside KSA	457	86.4
	Inside KSA	309	60.2
Source of medication in KSA	Pharmacist	117	36.6
	Physician	252	78.8
	Family/friend	8	2.5
Who handles your medication during Hajj?	Myself	424	81.2
	Family member	43	8.3
	Medical mission	94	18.0
Number of medications pilgrims currently using	1–4	472	91.6
	5–9	37	7.2
	≥10	6	1.2
Handling of remaining medication post-Hajj	Take it home	444	83.9
	Throw it away	81	15.3
	Other	6	1.1

KSA; Kingdom of Saudi Arabia.

**Table 4:** Medication storage during Hajj among pilgrims.

Medication Storage	Makkah *n* (%)	Madinah *n* (%)	Mina *n* (%)	Arafat *n* (%)
With self	448 (85.5)	409 (85.0)	413 (80.2)	415 (80.7)
With medical mission	82 (15.6)	72 (15.0)	109 (21.2)	106 (20.6)
In shared storage	12 (2.3)	8 (1.7)	5 (1.0)	4 (0.8)
Other	10 (1.9)	9 (1.9)	8 (1.6)	8 (1.6)

## Data Availability

Data available on request due to ethical restrictions.
